# Analysis of influencing factors of serum SCCA elevation in 309 CAP patients with normal CEA,NSE and CYFRA21-1

**DOI:** 10.3389/fonc.2024.1243432

**Published:** 2024-01-29

**Authors:** Jinghan Wang, Xiao Tang, Xin Liu, Jing Zhang

**Affiliations:** ^1^ Department of Clinical Laboratory, the Second Hospital of Dalian Medical University, Dalian, China; ^2^ Department of Health Statistics, School of Public Health, Dalian Medical University, Dalian, China; ^3^ Respiratory Department, the Second Hospital of Dalian Medical University, Dalian, China

**Keywords:** pneumonia, fever, SCCA, lung cancer, health education

## Abstract

**Introduction:**

Squamous cell carcinoma antigen (SCCA) is one of the auxiliary diagnostic indicators of lung squamous cell carcinoma, and an increase in serum SCCA can predict the occurrence of lung squamous cell carcinoma. However, whether SCCA is also elevated in pneumonia patients without malignancy is still not clear. Therefore, we studied influencing factors of elevated serum SCCA in patients with community-acquired pneumonia.

**Methods:**

We retrospectively enrolled 309 patients who were admitted to the Respiratory department with normal serum Carcinoembryonic antigen (CEA), Neuron specific enolase (NSE), and Cytokeratin 19 fragment (CYFRA21-1) level and were diagnosed with community-acquired pneumonia (CAP). The patients’ serum SCCA level, body temperature, age, sex, white blood cell (WBC) count, hypersensitive C-reactive protein (Hs-CRP) level, and serum amyloid A (SAA) were recorded. Logistic regression models were used to analyze the risk factors of SCCA elevation. The dose-response relationship between temperature and risk of SCCA increase was analyzed using Restricted cubic splines (RCS).

**Results:**

Of the 309 patients, 143(46.3%) showed elevated SCCA levels. The logistic regression analysis revealed a significant influence of age and body temperature on elevated SCCA *(P*<0.05) levels. For every one-year increase in age, the probability of elevated SCCA decreased by 3% [OR=0.97,95%CI:0.95,0.99].For every 1°C increase in body temperature, the risk of elevated SCCA increased by 2.75 times [OR=3.75,95%CI:2.55,5.49].The patients were sorted into quartiles based on body temperature. Compared with patients in the Q1 of body temperature group, patients in the Q3 group were at 7.92 times higher risk [OR=7.92, 95%CI:3.27,19.16].and the risk of elevated SCCA was increased by 22.85 times in the Q4 group [OR=23.85,95%CI:8.38,67.89] after adjusting for age, gender, Hs-CRP, SAA, and WBC. RCS analysis showed there was a linear relationship between temperature index and risk of elevated SCCA.

**Conclusion:**

In summary, for CAP patients with normal CEA,NSE and CYFRA21-1 level, age and body temperature are influencing factors of SCCA elevation. Higher body temperature has a strong association with the occurrence of SCCA elevation.

## Introduction

Lung cancer is one of the most common malignant tumors in the world. As the early symptoms of lung cancer are not obvious, most patients are diagnosed at an advanced stage, and the prognosis is poor (1). Pneumonia is a common benign disease in the respiratory department, and patients often have symptoms of fever, cough, sputum, hemoptysis and other clinical manifestations. Pneumonia can be cured with active anti-infective treatment. The early clinical symptoms of lung cancer patients are similar to pneumonia and lack specificity. For hospitalized pneumonia patients, in addition to anti-infective treatment, the exclusion of lung cancer has become one of the most important purposes of hospitalization for them.

SCCA is a tumor-specific antigen that was first discovered in the 1970s by Kato and Torigoe from cervical squamous cell carcinoma tissues (2). It is widely found in the cytoplasm of squamous cell carcinomas, such as the uterus, cervix, lung, head and neck, especially in nonkeratinized cancer cells, in which the content of SCCA is more abundant (3–6). SCCA exists in squamous epithelial cells is involved in the differentiation of squamous epithelium and tumor growth of tumor cells, and is often used in the diagnosis of squamous epithelium-derived carcinoma (7). SCCA is a specific marker of squamous cell carcinoma and is an independent prognostic factor for cervical squamous cell carcinoma (1).

As one of the tumor markers, SCCA alone does not confirm the tumor, but it can provide valuable information for the diagnosis and prognosis assessment of various malignant tumors, such as in the preconditioning evaluation of the tumor scope, evaluation of the treatment response and prediction of prognosis (4). As a tumor marker of various squamous cell cancers (esophageal cancer, lung cancer, head and neck cancer, anal canal cancer, cervical cancer, etc.),SCCA is of great significance in reflecting tumor stage, tumor size, interstitial infiltration, status of the lymphatic vascular space, and status of lymph nodes (8). Therefore, SCCA is particularly suitable for detecting squamous cell carcinoma (6, 7). Meanwhile, its elevation is also a poor prognostic factor for squamous cell carcinoma (4). Sun et al. (3) has shown that SCCA can initially evaluate the radiotherapy effect of lung cancer patients and has a certain predictive effect on prognosis. Yang et al. (1) has also pointed out that SCCA is important for the assessment of prognosis and survival assessment of lung cancer. An earlier article pointed out (9) that a high serum SCCA level is an independent poor prognostic factor in patients with peripheral squamous cell carcinoma.

For the past few years, there have been an increasing number of studies on SCCA in nonneoplastic diseases. In addition to tumors, elevated serum SCCA levels have also been detected in chronic liver disease, pulmonary infiltration with eosinophilia, renal insufficiency, and chronic inflammatory skin diseases (such as psoriasis, pemphigus, or eczema) (4, 8, 10–12). The serum SCCA level of diabetic nephropathy patients is significantly higher than that of normal proteinuria patients and healthy controls (10). A study has shown that the SCCA level is closely related to patients with varus papilloma, and SCCA may have the potential to be a useful biomarker for patients with varus papilloma (5).More articles have shown that SCCA is also elevated in some nonmalignant pulmonary diseases (2, 5). For example, there has been a case report showing that influenza B virus infection can lead to increased SCCA (13). Many studies have shown that the SCCA level also increases in bronchial asthma (13–15).

However, the elevation of SCCA in patients with pneumonia has not been studied. In clinical work, we found that some patients with pneumonia also had elevated serum SCCA, whereas other tumor markers, such as CEA, NSE, and CYFRA21-1,were normal, and a series of subsequent clinical examination could rule out the diagnosis of lung cancer. Therefore, in this study, we selected some patients whose CEA,NSE and CYFRA21-1 levels were normal and who had lung cancer excluded. We detected their SCCA levels, analyzed the relationship between SCCA concentration and body temperature, age and inflammatory indicators. The aim of the present study is to investigate the influencing factors of serum SCCA elevation in CAP patients with normal CEA,NSE and CYFRA21-1. Identification of these factors, which are helpful to improve the health education for CAP patients and reduce unnecessary examinations, is highly warranted.

## Methods

### Study subjects and exclusion criteria

A total of 1,300 patients diagnosed with pneumonia after admission to the Respiratory Department of the Second Affiliated Hospital of Dalian Medical University from January 2019 to December 2020 were considered for this study.

The patients included in the study were selected based on the following exclusion criteria:

(1) age ≥80 years and ≤15 years old;
(2) bronchial asthma;
(3) acute/chronic bronchitis, interstitial pneumonia, tuberculosis, bronchiectasis, and chronic obstructive pulmonary disease;
(4) patients with lung cancer and undiagnosed lung nodules;
(5) other malignant tumors;
(6) CEA,NSE, and CYFR21-1 tests were not performed during hospitalization;
(7) any of the serum CEA,NSE and CYFR21-1 test results was abnormal;
(8) pregnancy.

The diagnostic criteria of pneumonia were based on the Chinese Guidelines for Diagnosis and Treatment of Community-acquired Pneumonia (2016 edition) (16): 1. community disease onset; 2. showing the following related clinical manifestations of pneumonia: (i) recent cough, expectoration, or existing respiratory disease symptoms with or without purulent sputum/chest pain/dyspnea/hemoptysis; (ii) fever; (iii) pulmonary consolidation signs and/or wet rales; (iv) peripheral WBCs>10×109/L,OR<4×109/L with or without a neutrophilic left shift; and 3. chest imaging revealing new patchy infiltration, leaf/segment contrast, ground glass shadow, or interstitial changes with or without pleural effusion. Patients showing criteria 1 and 3 and any one of the 2 criteria were diagnosed with pneumonia, except for those with pulmonary tuberculosis, pulmonary tumor, noninfectious interstitial disease, pulmonary edema, atelectasis, pulmonary embolism, pulmonary eosinophil infiltration, and pulmonary vasculitis.

Ultimately, a total of 309 cases were enrolled, including 135 males and 174 females,143 with normal SCCA and 166 with elevated SCCA. According to the statistical rule, the number of cases with elevated SCCA was at least 10 times that of the included variables in the regression analysis. A total of 9 independent variables were included in this study, so the sample size requirement for statistical analysis was met.

### Measurement

For quantitative detection of serum SCCA, 3 mL of fasting venous blood was drawn, and a chemiluminescence immunoassay was performed using the MAGLUMI 2000 automatic chemiluminescence instrument. The immunoassay was performed with the reagents from the instrument’s supporting kit and operated in strict accordance with the manufacturer’s instructions.

The serum SCCA reference interval was 0 ng/mL–2.5 ng/mL. “SCCA elevation” refers to a serum SCCA level >2.5 ng/mL.

WBC count was measured by a Sysmex XN 9000 automatic hematology analyzer using flow cytometry, and other inflammation factors, such as Hs-CRP and SAA levels, were detected with a chemiluminescence immunoassay.

In this study, body temperature was measured by a thermometer reading, and “body temperature increase” refers to a body temperature >37.2°C. “Wheezing” refers to the auscultation of both lungs with rhonchi.

### Statistical methods

Patients with pneumonia were divided into two groups according to the SCCA detection value: the normal SCCA group (SCCA ≤ 2.5 ng/mL) and the elevated SCCA group (SCCA>2.5 ng/mL). The chi-square test was used to compare the differences between the enumeration data of two groups,i.e., age, sex, body temperature (normal/increased),and wheezing (yes/no). WBC, hs-CRP,and SAA data showed abnormal distribution expressed as the median M (P25,P75) and a comparison between the two groups was performed with a rank sum test of two independent samples.

Patients with pneumonia were grouped into quartiles from small to large according to SCCA levels. We used the chi-square test for categorical variables (age, sex, fever) and a multigroup rank sum test for group comparisons of continuous-type variables. Logistic regression was used twice to analyze the risk factors for SCCA elevation. First, the independent variables selected were all the observed indicators to determine risk factors. Second, body temperature was divided into quartiles, and risks of SCCA elevation for each quartile were evaluated by setting the lowest quartiles of body temperature as the reference group. Model 1 was adjusted for age and gender and BMI, and model 2 was further adjusted for Hs-CRP, SAA, and WBC. Restricted cubic spline (RCS) analysis was applied to analyze the dose–response relationship between the temperature and the risk of elevated SCCA.

Statistical analysis was performed using SPSS 26.0. *P*<0.05 was considered significant. The RCS analysis was performed in R 4.0.3.

## Results

### Patient demographics

Ultimately, 309 CAP patients,135 males and 174 females, whose serum CEA, NSE, and CYFRA21-1 levels were normal met our enrolment criteria.

For analysis, the patients were grouped based on the level of serum SCCA and body temperature. The serum SCCA level was ≤2.5 ng/mL in 143 (46.28%) patients and >2.5 ng/mL in 166 (53.72%) patients. Among the 309 patients,125 (40.45%) had a normal body temperature, and 184 (59.55%) had a fever.

### Comparison of basic conditions and inflammatory indicators of pneumonia patients in two groups

Patients in the elevated SCCA group were younger than those in the normal SCCA group but had a higher probability of wheezing and increased body temperature (*P*<0.05). Moreover, the inflammation indices, such as Hs-CRP and SAA, were significantly higher than those in the normal group (*P*<0.05) (**Table 1**). There was no significant difference between sex and WBC count in the two groups.

**Table 1:** General Characteristics of participants.

Indices	Normal SCCA (N=166)	Elevated SCCA (N=143)	Z value	*P* value
Age	60 (46,65)	50 (33,63)	3.96	*<0.05*
Gender N(%)				
Male	65 (39.2%)	70 (49.0%)	2.99	0.083
Female	101 (60.8%)	73 (51.0%)		
Wheezing N(%)				
Yes	135 (81.3%)	130 (90.9%)	5.78	*<0.05*
No	31 (18.7%)	13 (9.1%)		
Body temperature N(%)				
Normal	107 (64.5%)	18 (12.6%)	85.81	*<0.05*
Increase	59 (35.5%)	125 (87.4%)		
WBC (*10^9/L)	7.70 (6.09, 10.24)	7.83 (5.82, 9.44)	1.23	0.220
Hs-CRP (mg/L)	11.69 (2.03, 44.98)	33.70 (13.40, 74.55)	4.82	*<0.05*
SAA (mg/L)	31.46 (9.33, 177.10)	179.38 (47.95, 237.27)	4.97	*<0.05*

Data are the mean number (percentage) or median (P_25_,P_75_).

### Comparison of patient characteristics according to serum SCCA levels

The patient groups were ranked from small to large according to SCCA levels. The top 25% of patients were in Group 1,the patients in the top 25%–50% were Group 2,the patients in the top 50%–75% were in Group 3,and the patients in the top >75% were in Group 4.

Our analysis showed statistically significant differences in age, fever, Hs-CRP, and SAA levels among the different groups. Patients in Group 4 had the highest serum SCCA level, while their age was significantly lower. In contrast, the proportions of patients with fever and the Hs-CRP, and SAA levels were significantly higher than those in the other three groups (*P*<0.05) (**Table 2**). The trend increased with the SCCA level. No significant difference was found among the four groups between wheezing (*P*=0.075) and WBC count (*P*=0.103).

**Table 2:** Comparison of participants’ characteristics with the serum SCCA levels.

Indices	SCCA,ng/ml				Z value	*P* value
	Q1 (<33.00)	Q2 (33.00-50.00)	Q3 (50.00-63.00)	Q4 (>63.00)		
Age	60 (48, 67)	60 (45, 65)	60 (45, 65)	50 (32, 64)	18.36	*<0.001*
Wheezing N(%)						
Yes	64 (80%)	61 (81.3%)	60 (89.6%)	80 (92.0%)	6.89	0.075
Temperature (°C)	36.8 (36.7, 37.7)	36.8 (36.7, 38.0)	38.4 (37.4, 39.0)	38.9 (38.0, 39.3)	85.64	*<0.05*
Fever N (%)						
Yes	24 (30.0%)	30 (40.0%)	51 (76.1%)	79 (90.8%)	83.82	*<0.05*
WBC(*10^9/L)	7.46 (5.85, 9.71)	8.31 (6.00, 11.29)	7.87 (6.52, 9.86)	7.52 (4.98, 8.98)	61.19	0.103
Hs-CRP (mg/L)	8.57 (1.56, 31.55)	15.64 (2.12, 55.07)	31.38 (8.81, 72.65)	34.50 (13.61, 74.58)	22.06	*<0.05*
SAA (mg/L)	29.29 (9.22, 163.03)	45.66 (9.07, 176.05)	160.00 (23.02, 222.31)	192.02 (79.47, 255.13)	26.98	*<0.05*

Data are the mean number (percentage) or median (P_25_,P_75_).

### Factors impacting elevated SCCA levels

Logistic regression was used for the first analysis of risk factors for increased SCCA. The results showed that age and body temperature were associated with elevated SCCA levels in all the observed measures (*P*<0.05). Age was a protective factor against SCCA elevation, while body temperature was a risk factor for SCCA elevation. The probability of SCCA increasing decreased by 3% with an age increase of one year. In contrast, the risk of SCCA increased by 2.75 times with a temperature increase of 1°C (**Table 3**).

**Table 3:** The risks factors of elevated SCCA levels by a logistic model.

Indices	B value	OR (95% CI)	*P* value
Age	-0.028	0.97 (0.95, 0.99)	*<0.05*
Gender (male/female)	-0.594	0.52 (0.29, 1.05)	0.071
Wheezing (Yes/No)	-2.400	0.79 (0.31, 1.99)	0.613
Temperature (°C)	1.320	3.75 (2.55, 5.49)	*<0.05*
WBC (*10^9/L)	-0.088	0.92 (0.82, 1.02)	0.104
Hs-CRP (mg/L)	-0.005	0.99 (0.99, 1.01)	0.368
SAA (mg/L)	0.001	1.00 (0.99, 1.00)	0.682

### Effects of body temperature on serum SCCA elevation

**Figure 1:**
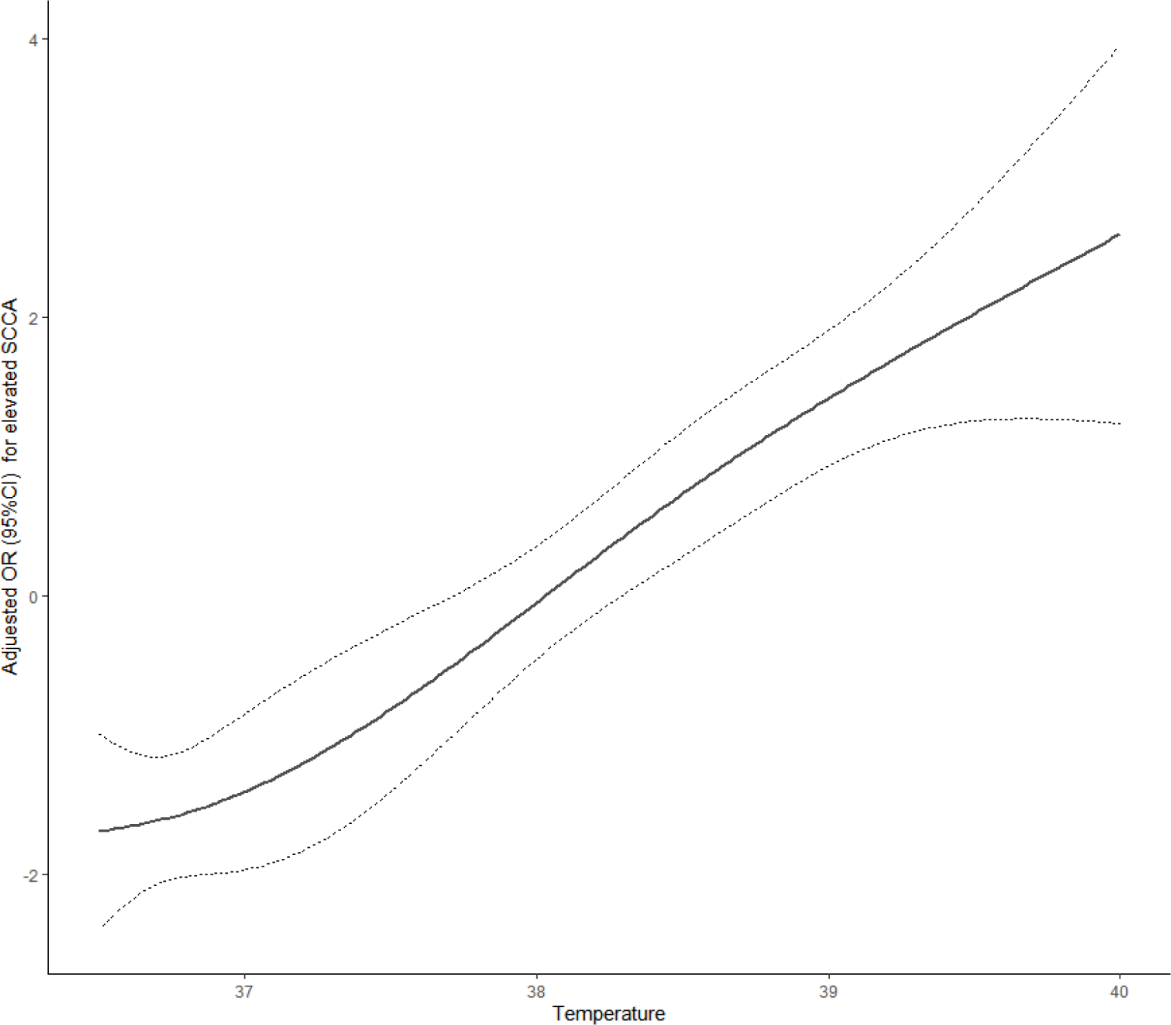
Dose-response relationship between temperature index and SCCA.

For the second step of regression analysis, body temperature was divided into four groups as an independent variable for analysis. The logistic regression results showed that the risk of SCCA value elevation was 7.92 times higher in Q3 patients than that in Q1 patients (*P*<0.05) and the risk of SCCA elevation in Q4 patients was 22.85 times higher than that in Q1 patients (*P*<0.05). For every 1°C increase in body temperature, the risk of elevated SCCA increased by 2.75 times[OR=3.75,95%CI:2.55,5.49] (**Table 4**). The RCS analysis showed a linear relationship between the temperature index and the risk of elevated SCCA (Pnonlinear =0.7371, **Figure 1**).

**Table 4:** The association of body temperatures with SCCA elevation by logistic regression analyses.

Indices	Model 1			Model 2		
	B value	OR (95% CI)	*P* value	B value	OR (95% CI)	*P* value
Body temperatures (continuous)	1.29	3.63 (2.69, 4.88)	*<0.05*	1.32	3.75 (2.55, 5.49)	***<0.05***
Quartiles of body temperatures						
Q1 (≤36.7°C)	–	–	–	–	–	–
Q2 (36.8°C-37.9°C)	-0.01	0.99 (0.43, 2.26)	0.98	0.38	1.46 (0.60, 3.56)	0.40
Q3 (38.0°C-38.8°C)	2.01	7.42 (3.55, 15.53)	*<0.05*	2.07	7.92 (3.27, 19.16)	*<0.05*
Q4 (38.9°C-42.0°C)	3.09	21.86 (9.22, 51.81)	*<0.05*	3.17	23.85 (8.38,67.89)	*<0.05*

Model 1: Adjusted for: age and gender.

Model 2: Adjusted for: age, gender, Hs-CRP, SAA, and WBC.

## Discussion

SCCA is originally purified from cervical squamous cell carcinoma (17, 18) and it is widely expressed in tongue, tonsil, esophagus, cervix, vagina, trachea, skin and other normal tissues (14, 17, 19). SCCA can be used as a diagnostic marker for cervical cancer, lung cancer, esophageal cancer, head and neck cancer and other squamous cell carcinomas (8, 14, 17, 18). Studies have shown that the sensitivity of SCCA in the diagnosis of non-small cell lung cancer (NSCLC) is 17%,and the specificity can reach 95%. The sensitivity and specificity of diagnosis in lung squamous cell carcinoma are 95% and 32% (20). Ando et al. measured seven serum biomarkers in 312 NonSmall Cell Lung Cancer (NSCLC) patients and found that SCCA had the highest positive rate at 55.4%,followed by CYFRA 21-1 (48.2%) (4). Clinically, SCCA is often used in combination with CYFRA 21-1,CEA,and NSE to screen for early stage of lung cancer. In recent years, a few studies and case reports have found that SCCA levels are elevated in benign lung diseases, such as asthma (8, 14, 15, 21), influenza virus infection (13), respiratory syncytial virus infection (22),tuberculosis (23), pulmonary sarcoidosis (23), pulmonary fibrosis (24), critically ill patients with COVID-19 (25), eosinophilic pulmonary infiltration (4, 26) and other diseases. The mechanism of the serum SCCA increased in patients with allergic diseases has been extensively studied (8, 27). For example, in atopic dermatitis and bronchial asthma patients, increased SCCA is caused by IL-4 and IL-13, which are secreted by Th2 cells (15, 17, 28, 29). These cytokines act on skin keratinocytes or bronchial epithelial cells (8), inducing high expression of SCCA in airway epithelial cells and/or keratinocytes.

The present study is a retrospectively clinical investigation of the serum SCCA levels of 309 CAP patients with normal serum CEA,CYFRA21-1 and NSE. The impact factors of SCCA elevation was analyzed by grouping the patients into quartiles according to SCCA and body temperature, respectively. To the best of our knowledge, this study may be the first to report that body temperature and age influence the elevation of serum SCCA in patients with community-acquired pneumonia (CAP). The main finding of the study was that age was a protective factor against SCCA increase, while body temperature was a risk factor for SCCA value elevation. There was a linear relationship between temperature index and risk of elevated SCCA.

Why does body temperature elevation lead SCCA increase in CAP patients? It is well known that fever, as an important host defense mechanism, is accomplished by integrated physiological and neural circuits (30). Transient receptor potential cation channel subfamily M member 8 (TRPM8) is a kind of cold-sensing neuron expressed in the nerve endings of sensory neurons and keratinocytes in the epidermis of skin. Its activation induces a series of cold defenses, such as brown adipose tissue thermogenesis, shivering thermogenesis and skin vasoconstriction (31). As the outermost barrier tissue of human body, skin is composed of epidermis, dermis and subcutis (32). Meanwhile, as a major immune organ, skin contains a large number of type 2 innate lymphoid cells (ILC2s) (32, 33). ILC2s in the dermal skin are activated by signals from cold-sensing neurons (31), which respond to the changes in ambient temperature to help regulate thermal homeostasis in the skin. It has been reported that the activation of TRPM8 can promote thermogenesis, and dermal ILC2s are activated by stimulating TRPM8 (31). The study also found that TRPM8 expressed in dermis was partially responsible for the activation of skin-resident ILC2s,and TRPM8,which is expressed in epidermal keratinocytes, may also be involved in sensing ambient temperature to promote local ILC2s activation (31). ILC2s express the transcription factors GATA 3 and RORα during development which are capable of producing type 2 cytokines such as IL-4,IL-5,and IL-13 (31, 34). Stimulated by interleukin-4 (IL-4) or interleukin-13 (IL-13), keratinocytes can secrete SCCA1 and SCCA2 (17, 21).

Thus, we speculate when a CAP patient had a fever, the skin cold receptors could activate skin-resident ILC2s to secrete IL-4,IL-5 and IL-13.Then,the cytokines trigger keratinocytes to secrete SCCA.

Furthermore, does SCCA directly mediate the inflammatory response to pneumonia? Studies showed that SCCA could also be detected in the serum of patients with lung diseases such as bronchitis and pneumonia (35). These findings raise the possibility that SCCA may act as a marker for certain inflammation. A paper also mentioned that the Clade B of serine protease inhibitors family 3(SERPINB3)and the Clade B of serine protease inhibitors family 4 (SERPINB4), also known as squamous cell carcinoma antigen-1 and -2(SCCA1/2). Elevated levels of these inhibitors were detected during inflammation, which may indicate that they are upregulated to help suppress the inflammatory response. Similarly, their overexpression may indicate that they can cause a pro-inflammatory response (36). In our study, the inflammation indicators, such as Hs-CRP and SAA, were significantly higher than those in the normal group (P<0.05).However, in the Logistic regression analysis of risk factors for increased SCCA, the P values of them did not reach the statistical significance (P= 0.065).Therefore, we consider that SCCA might be involved in mediating the inflammatory response in patients with pneumonia, but we have not conducted relevant experiments and in-depth research analysis, and more studies are needed to confirm this situation in the future.

Interestingly, this study also found that, unlike the effect body temperature, increase of age was a protective factor for SCCA elevation. It means that the risk of SCCA elevation decreases with increase of age. We speculate that it might be related with skin aging. The skin exerts its barrier function through a multilayered structure comprised of three distinct anatomical compartments: epidermis, dermis, and subcutis (32). Keratinocytes exist in the epidermis (37) and ILC2s are distributed in the dermis (32, 33). Considered both physiologic and inevitable, skin aging is a degenerative phenomenon (38). Studies (39) have shown there is a functional difference in the stratum corneum of young versus old skin because recovery of aged skin from insults to this layer are significantly slower than those seen in young skin, and permeability to certain substances is altered. We suggested that the increased risk of SCCA decreased associated with age may be due to the decline in secretory function of skin cells with age increasing. That’s just our conjecture, we need to expand the sample size and data sources, reduce confounding bias, and conduct more in-depth studies.

There are some limitations in our study. First, 309 patients were enrolled in this study, of which 143 were in the SCCA elevated group. Although the sample size was small, the sample size met the statistical requirements. It is undeniable that insufficient sample size may introduce significant interference in the use of statistical models with numerous covariates. Since this is a single-center study, it is necessary to conduct multi-center studies in the future to expand the sample size and make the conclusions more reliable. Second, for CAP patients, we did not further group them by pathogen to analyze whether they were associated with increased SCCA. It is because the positive rate of pathogenic examination is generally low, and the amount of pathogen examination performed in our patients is small. This information cannot be obtained. Third, we do not conduct the in-depth studies on SCCA whether or not mediates inflammation. We can design more experiments in the future to explain this question.

In summary, we find that for CAP patients with normal serum CEA, NSE, and CYFRA21-1, body temperature and age are significantly correlated with increased SCCA. Increased body temperature is a risk factor for SCCA elevation, while increasing age is a protective factor for SCCA elevation. For CAP patients with elevated SCCA, appropriate explanations can be done to reduce the anxiety of patients and their families about lung cancer.

## Data availability statement

The raw data supporting the conclusions of this article will be made available by the authors, without undue reservation.

## Ethics statement

The studies involving humans were approved by the Second Affiliated Hospital of Dalian Medical University. The studies were conducted in accordance with the local legislation and institutional requirements. Written informed consent for participation in this study was provided by the participants’ legal guardians/next of kin.

## Author contributions

JZ and JW collected data, reviewed literature, and wrote manuscripts. XT was responsible for the statistical analysis. XL was in charge of data collection, literature review. All authors read and approved the final manuscript.
